# Plasma Circulating Tumor DNA Sequencing Predicts Minimal Residual Disease in Resectable Esophageal Squamous Cell Carcinoma

**DOI:** 10.3389/fonc.2021.616209

**Published:** 2021-05-20

**Authors:** Tao Liu, Qianqian Yao, Hai Jin

**Affiliations:** ^1^ Department of Thoracic Surgery, Changhai Hospital, Second Military Medical University, Shanghai, China; ^2^ Department of Medical Science, Shanghai AccuraGen Biotechnology Co., Ltd., Shanghai, China

**Keywords:** esophageal squamous cell carcinoma, circulating tumor DNA, next-generation sequencing, minimal residual disease, prognostic marker

## Abstract

Esophageal squamous cell carcinoma (ESCC) is lethal as tumors are rarely detected at an early stage and have a high recurrence rate. There are no particularly useful biomarkers for the prognostic prediction of ESCC. Circulating tumor DNA (ctDNA) is becoming an important biomarker for non-invasive diagnosis and monitoring tumor prognosis. Here, we aimed to analyze variations in plasma cell-free DNA (cfDNA) amount to search for minimal residual disease (MRD). Plasma and white blood cells (WBCs) of 60 patients were collected before tumor resection and a week after surgery. Tumor specimens were also collected as formalin-fixed paraffin-embedded (FFPE) samples. All samples were extracted to analyze the genetic alterations of 61 genes using capture-based next-generation sequencing (NGS). Tumor variants were detected in 38 patients with ESCC, and the two driver genes with the highest mutation frequency were *TP53* and *PIK3CA*. Of the pre-surgical plasma cfDNA samples, 73.7% of identified variants matched the tissue. In patients who did not receive adjuvant therapy after surgery, postoperative cfDNA-positive patients had shorter overall survival (hazard ratios (HR), 25.8; 95% CI, 2.7–242.6; P = 0.004) and were more likely to relapse than postoperative cfDNA-negative patients (HR, 184.6; 95% CI, 3.6–9576.9; P = 0.01). Detection of ctDNA after surgical tumor excision is associated with tumor relapse and disease-specific survival, and can be used as a prognostic biomarker for MRD detection in ESCC.

## Introduction

Esophageal cancer is lethal and is the sixth leading cause of cancer-related death (1), in which esophageal squamous cell carcinoma (ESCC) accounts for approximately 90% of cases (2). The preferred treatment strategy for locally advanced disease includes neoadjuvant therapy plus surgery (3); however, relapse following complete resection of ESCC remains common. It has been reported that 40% to 50% of patients with supposedly localized esophageal cancer suffer from recurrence or metastasis within two years after surgery (4–6), and the median survival after recurrence is only 8 months (7). Nevertheless, even in early-stage patients, relapse can occur within two years after surgery. A reason for early relapse in ESCC patients might be occult minimal tumor cell dissemination already present at the time of surgery, which is undetectable by current clinical radiologic and routine histopathological methods (6). Therefore, predicting recurrence when tumors are still minimal or occult may enhance the survival rates of patients with ESCC (8). Accurate identification of biomarkers to assess tumor burden and early detection of tumor recurrence is critical to ensure timely and effective therapy (9).

Currently, several prognostic factors have been accepted for clinical application, such as lymph node status and tumor stage; however, disease status and clinicopathological status cannot clearly identify which patients have a higher risk of recurrence (7, 10). Moreover, protein biomarkers have limited sensitivity and specificity, and their expression levels are easily affected by other factors. Therefore, there remains a need to identify better prognostic markers that can be used in biological fluids (11).

Circulating tumor DNA (ctDNA) in the blood is small DNA fragments released upon apoptosis or necrosis of primary and metastatic tumor cells (12, 13). The short half-life of cfDNA endows it with the potential to dynamically monitor changes in tumor burden (14). In recent years, ctDNA detected using next-generation sequencing (NGS) has been used to assess the presence of minimal residual disease (MRD), thereby predicting the risk of disease recurrence in a variety of solid tumors, such as lung cancer (15), breast cancer (16), and colorectal cancer (17). However, there are relatively few studies on ctDNA in ESCC.

In this study, tumor and matching plasma samples from 60 esophageal patients were collected and sequenced using a capture-based 61 gene panel. Our research aims to explore the utility of NGS-based cfDNA analysis to identify biomarkers with prognostic information on recurrence and survival.

## Materials and Methods

### Subjects Enrollment

This study took place in the Department of Thoracic Surgery, Changhai Hospital, Shanghai, from August 2015 to December 2016. The inclusion criteria were as follows: Pathologically confirmed patients with ESCC who 1) underwent surgery; 2) did not undergo any anti-tumor treatment before the first blood collection; 3) had no history of other malignancies. The study was conducted in accordance with the Declaration of Helsinki Principles and approved by the Institutional Review Board of Changhai Hospital. Informed consent was obtained from all subjects.

**Figure 1:**
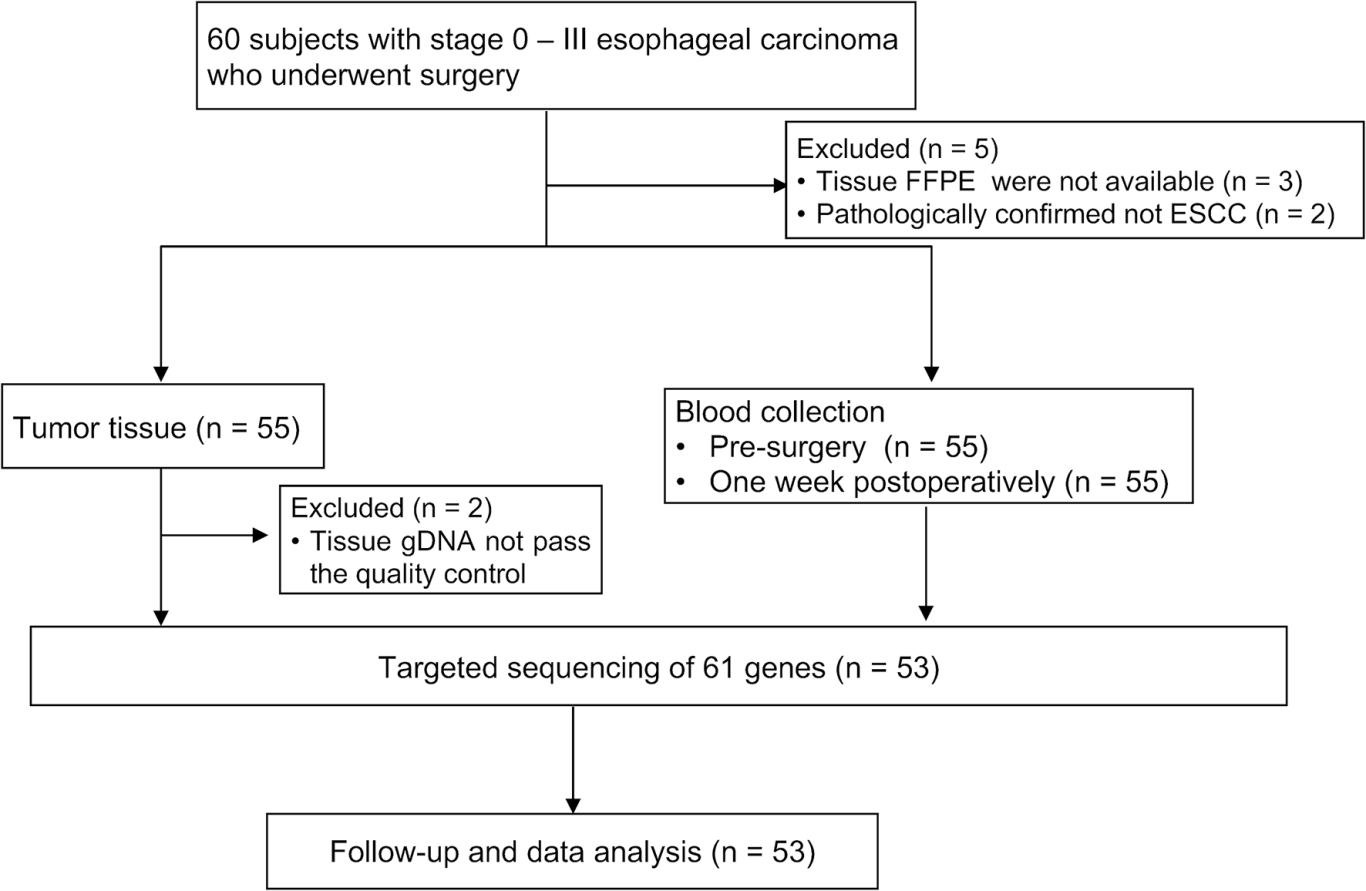
Overview of patient enrollment and sample collection.

Sixty patients were initially enrolled; two patients who were subsequently diagnosed with adenosquamous esophageal carcinoma or esophageal small cell carcinoma, three patients whose FFPE samples were not available, and two patients with insufficient tissue samples were excluded from the study (**Figure 1**). Surgery was performed *via* the Sweet, Ivor-Lewis, or McKeown procedure with/without lymphadenectomy.

### Sample Collection and DNA Extraction

Tumor tissue samples were collected from patients with ESCC *via* surgery, and FFPE sections were prepared by the pathology department according to the standard procedures of our hospital (18). For each patient, 10 ml of venous blood sample was obtained at two time points, the day before surgery and one week post-surgery, in plastic K_2_EDTA tubes (BD, Franklin Lakes, NJ, USA). Blood samples were centrifuged at 1,900 ×g for 10 min at 4°C, and the upper phase of plasma and white blood cells (WBCs) from the middle phase were collected in new Eppendorf tubes. The upper plasma was further centrifuged at 4°C and 16,000 ×g for 10 min to pellet the remaining cells. All tubes were labeled with the matching patient ID and the sample collection date, and then immediately stored at −80°C until DNA extraction. The time interval between sample collection and freezing was 4 h.

cfDNA was isolated from 3 to 5 ml plasma using the QIAamp Circulating Nucleic Acid Kit (QIAGEN, Hilden, Germany) following the manufacturer’s protocol. cfDNA concentration was measured using a Qubit fluorometer 3.0 (Life Technologies, Grand Island, NY) and the cfDNA fragments were quantified using a 2100 bioanalyzer (Technologies, Palo Alto, CA). Genomic DNA (gDNA) was extracted from WBCs or FFPE samples using the QIAamp DNA Blood Mini Kit (QIAGEN) and TIANquick FFPE DNA Kit (TIANGEN, Beijing, China) according to the manufacturer’s instructions. Isolated cfDNA and gDNA were stored at −80°C until use.

### NGS of gDNA and cfDNA

Plasma cfDNA, gDNA from FFPE specimens and WBCs were all sequenced using capture-based targeted NGS (19). All sequencing was performed at the Shanghai Yunsheng Medical Laboratory Co., Ltd. Briefly, gDNA was sonicated into fragments with a peak fragment size of approximately 200 bp. After sonication, 100 ng of fragmented gDNA was used for standard NGS library construction using KAPA library preparation kit (KAPA Biosystems, Boston, MA, USA) following the manufacturer’s protocol. The gDNA NGS library was captured using a panel consisting of 61 cancer-related genes (19) of clinical significance, followed by sequencing with 101 bp paired-end runs on an Illumina HiSeq 2500 (Illumina, San Diego, CA, USA). Generally, sequence reads collected from HiSeq were aligned to the hg19/GRCh37 reference genome using the Burrows-Wheeler Alignment tool (20). Duplicate removal, single nucleotide variants, and indel calling were performed using VarDict (21). Variant annotation and filtering were based on the GEMINI analysis (22). Somatic mutations specific to tumors were noted when they met all the following criteria: 1) Not detected in the gDNA of white blood cells; 2) not present in the 1000G data; 3) mutant allele frequencies (MAF) > 5%; 4) at a locus with depth coverage of >200 both in FFPE and WBC gDNA.

cfDNA analysis was also carried out using the Accu-Act panel. Firefly NGS assay based on circularized and tandem error correction cfDNA technology (Yunsheng Medical Laboratory, Shanghai, China) was performed as previously reported (19). NGS libraries were sequenced on an Illumina HiSeq 2500, and unique sequencing reads were identified using the Firefly proprietary algorithm. WBC gDNA sequencing data were used to filter germline mutations and clonal hematopoietic mutations. For candidate cfDNA variant detection, the following criteria were applied: 1) No germline or clonal hematopoietic mutations; 2) not present in the 1000G data; 3) MAF > 0.02%; 4) variant-supporting consensus reads of ≥3.

### Statistical Analysis

Disease-free survival (DFS) was determined from the time of surgery to cancer relapse or the last follow-up, and overall survival (OS) was defined as the time from surgery to death or the last follow-up. Median DFS and OS and 95% confidence intervals (95% CI) were observed using the Kaplan-Meier method, and survival curves of different groups were analyzed using the log-rank test. Cox proportional hazards model was used to identify factors affecting prognosis, and it was used to create survival hazard ratios (HRs). The level of statistical significance was set as two-sided at p < 0.05. Statistical analysis was performed using SPSS 26.0 software package (IBM Corp., Armonk, NY) and figures were established using version 8.0 GraphPad Prism (GraphPad Software, San Diego, CA).

## Results

### Clinicopathologic Characteristics

**Figure 2:**
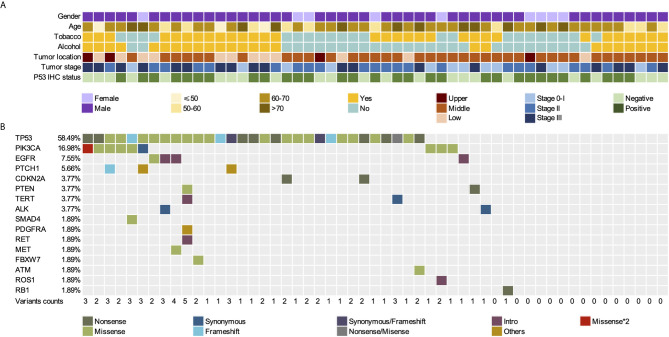
Somatic mutations in FFPE tissues of 53 patients with ESCC. **(A)** Gender, age, tumor location, tumor stage, and p53 IHC status of 53 patients. **(B)** Mutation landscape of FFPE samples. FFPE, Formalin-Fixed Paraffin-Embedded; ESCC, esophageal squamous cell carcinoma; IHC, immunohistochemistry.

Patient enrollment and study overview are described in **Figure 1**; 53 subjects were eligible for data analysis. The patient and pathological characteristics are presented in **Table 1** and **Figure 2A**. The median age at the time of diagnosis was 65 years (range, 46–79 years), 44 (83.02%) patients were male, and nine (16.98%) were female. Patients with a history of both smoking and drinking accounted for 41.51%, with the remaining patients with a history of smoking (16.98%) or drinking (5.66%) or non-smokers and non-drinkers (35.85%). Based on the 8th edition of the AJCC/UICC staging system, tumor stages 0 to I, II, and III were represented as follows: 22.64% (N = 12), 41.51% (N = 22), and 35.85% (N = 19), respectively. Most tumors (69.81%) originated from the middle thoracic esophagus, 71.70% was medium differentiated, 24.53% showed angiolymphatic invasion, and 81.13% showed neurological invasion. The median value of the maximum tumor diameter was 3.5 cm (range, 0.9–9 cm) (**Supplementary Table S1**). *TP53* expression was detected in 56.60% of patients using immunohistochemistry (IHC). None of the enrolled patients received neoadjuvant therapy, and 19 (35.85%) patients received adjuvant treatment.

**Table 1:** Patient characteristics.

Variable		Case (n = 53)	Percentage
**Age**			
	Median (range)	65 (46–79)	
**Gender**			
	Male	44	83.02%
	Female	9	16.98%
**Tobacco and alcohol use**			
	Both	22	41.51%
	Tobacco only	9	16.98%
	Alcohol only	3	5.66%
	None	19	35.85%
**TNM stage**			
	0 - I	12	22.64%
	II	22	41.51%
	III	19	35.85%
**Tumor location**			
	Upper	4	7.55%
	Middle	37	69.81%
	Lower	12	22.64%
**Tumor differentiation**			
	G1	5	9.43%
	G2	38	71.70%
	G3	7	13.21%
	NA*	3	5.66%
**Angiolymphatic invasion**			
	Yes	13	24.53%
	No	40	75.47%
**Neurological invasion**			
	Yes	43	81.13%
	No	10	18.87%
**Maximum tumor diameter**			
	Median (range)	3.5 (0.9–9)	
**Surgery procedure**			
	Sweet	4	7.55%
	Ivor lewis	40	75.47%
	Mckeown	9	16.98%
**P53 status**			
	Positive	30	56.60%
	Negative	23	43.40%
**Adjuvant therapy**			
	Chemotherapy	7	13.21%
	Radiochemotherapy	6	11.32%
	Radiotherapy	6	11.32%
	No	29	54.72%
	NA*	5	9.43%

*NA, not available.

### Mutational Landscape of ESCC in FFPE and Pre-Surgical cfDNA

Tumor gDNA in FFPE, as well as pre-surgical and post-surgical plasma cfDNA, of 53 patients with ESCC were analyzed using Accu-Act NGS (**Supplementary Table S2**). The average sequencing depth of FFPE, WBCs, pre-surgical cfDNA, and post-surgical cfDNA were 528×, 877×, 9274×, and 8482×, respectively. A total of 71.7% (38/53) of the ESCC tissue specimens showed at least one mutation. Seventy-one tumor-specific somatic mutations among 16 genes in 38 FFPE samples were identified, including genes with recurrent somatic alterations, such as *TP53* (58.49%), *PIK3CA* (16.98%), *EGFR* (7.55%), *PTCH1* (5.66%). *PTCH1*, and *TP53* mutations often co-occurred, and 15 FFPE specimens did not have any mutations (**Figure 2B**). *TP53* and *PIK3CA* mutations were present in 58.49% (31/53) and 16.98% (9/53) of the tissue samples, respectively. Comparison of *TP53* and *PIK3CA* mutation status between tissues and cfDNA is summarized in **Supplementary Table S3**. Among *TP53* or *PIK3CA* mutant tumors, the cfDNA mutation detection sensitivity was 71.0% (22/31) for *TP53* and 66.7% (6/9) for *PIK3CA*. The overall sensitivity of detecting the mutations on these two genes in cfDNA was 70.0% (**Supplementary Table S4**). No significant difference was found in the mutation frequency of TP53 and PIK3CA between different smoking and drinking groups (**Supplementary Table S5**).

### Post-Surgical cfDNA Status and Prognosis

In our study, three subjects could not be followed up, five patients had unknown DFS, and 21 recurrence and 20 death events due to tumor progression were investigated. The follow-up time ranged from 1.5 to 50.67 months, with a median of 34.8 months. We used tumor-specific mutation as a molecular marker, and 38 subjects were successfully stratified by postoperative ctDNA-positive (ctDNA+) and negative (ctDNA-) ctDNA status. Six of these 38 patients (15.8%) had tumor-specific mutations in their post-surgical plasma (ctDNA+). **Table 2** summarizes the variants and MAFs identified in ctDNA+ cases. Six ctDNA+ subjects developed five relapse and death events, with a relapse rate of 83.3% and a mortality rate of 83.3%.

**Table 2:** Tumor-specific variants of post-surgical plasma cfDNA.

Patient ID	Gene	Amino acid variation	Nucleotide alteration	Chromosome alteration	Tumor FFPE gDNA MAF	Pre-surgical cfDNA MAF	Post-surgical cfDNA MAF
CHE004	FBXW7	FBXW7:p.D440N	NM_033632.3:c.1318G>A	chr4:g.153249460C>T	61.60%	2.14%	0.59%
CHE019	PTEN	PTEN:p.R130Q	NM_000314.6:c.389G>A	chr10:g.89692905G>A	15.53%	5.72%	0.18%
	TP53	TP53:p.R273H	NM_000546.5:c.818G>A	chr17:g.7577120C>T	34.95%	4.05%	0.87%
	PDGFRA	PDGFRA:p.?	NM_006206.4:c.*12G>A	chr4:g.55161451G>A	17.16%	2.96%	0.10%
	TERT	NA	NM_198253.2:c.2654+10G>A	chr5:g.1266569C>T	7.03%	1.05%	0.24%
CHE021	PTCH1	NA	NA	chr9:g.98278972T>C	71.49%	44.24%	39.03%
CHE024	TP53	TP53:p.E294*	NM_000546.5:c.880G>T	chr17:g.7577058C>A	52.96%	8.35%	3.79%
CHE036	TP53	TP53:p.W146*	NM_000546.5:c.438G>A	chr17:g.7578492C>T	8.58%	4.07%	4.31%
CHE041	PIK3CA	PIK3CA:p.H1047R	NM_006218.3:c.3140A>G	chr3:g.178952085A>G	37.67%	0.62%	0.04%

cfDNA, cell-free DNA; gDNA, genomic DNA; MAF, mutant allele frequency.

To eliminate the effect of adjuvant therapy, we explored the ability of postoperative ctDNA status to predict recurrence in patients with ESCC who did not receive adjuvant treatment. Of the 38 patients with tumor-matching cfDNA variants, 60.5% (23/38) did not receive adjuvant therapy, and ctDNA was detected postoperatively in four of 23 patients (17.4%), relapse was investigated in three of four patients (75%), and death occurred in all four cases (100%). The remaining 19 of 23 (82.6%) subjects were postoperative ctDNA-, where disease recurrence was recorded in two (10.5%) patients, with one documented death. cfDNA variants were present in the blood plasma of patient #3 before surgery, with only one matching mutation (*PIK3CA*: p.Y644H, 2.78%) in the postoperative blood, while the variant was not detected in the tumor. For patient #17, pre-surgical cfDNA analysis showed *GNAQ* p.F220L (0.36%) and *PIK3CA* p.A598 (3.9%) variants while these mutations were not detected in the FFPE tumor sample, and the MAF of the two alterations was 0.1% and 2.32%, respectively (**Supplementary Table S6**).

**Figure 3:**
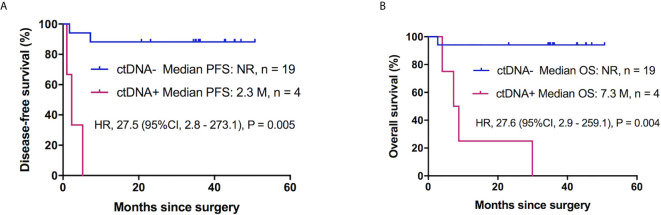
Postoperative ctDNA mutation status and prognosis of patients with ESCC without adjuvant therapy. **(A)** Kaplan-Meier survival curves for DFS analysis between postoperative ctDNA+ and ctDNA- patients. **(B)** Kaplan-Meier estimates of OS according to postoperative ctDNA status in patients with ESCC without adjuvant chemotherapy. ESCC, esophageal squamous cell carcinoma; ctDNA, circulating tumor DNA; DFS, disease-free survival; OS, overall survival, NR, not reach.

In this cohort, we also explored the correlation between the clinical risk factors associated with patient prognosis. Clinicopathological variables were not significantly associated with DFS, whereas tumor location, tumor stage, and tumor angiolymphatic invasion were significantly associated with OS in univariate analysis (**Table 3**). Postoperative ctDNA status had a more notable impact on DFS and OS than any other clinicopathological risk factor. Patients with postoperative ctDNA+ status had a markedly shortened DFS compared to those with ctDNA- status (HR, 27.5; 95% CI, 2.8–273.1; P = 0.005, **Figure 3A**). The OS of patients with postoperative ctDNA+ status was 7.30 months whereas that of those with postoperative ctDNA- status was not reached (HR, 27.6; 95% CI, 2.9–259.1; P = 0.004; **Figure 3B**). After a multivariable adjustment, postoperative cfDNA+ patients were more likely to relapse than postoperative cfDNA- patients (HR, 184.6; 95% CI, 3.6–9576.9; P = 0.01), and the OS was also shortened (HR, 25.8; 95% CI, 2.7–242.6; P = 0.004).

**Table 3:** Prognosis analysis by clinicopathological variables and postoperative ctDNA status in patients not treated with adjuvant therapy.

Parameter	*Univariate analysis*				*Multivariate analysis*			
	DFS		OS		DFS		OS	
	HR	P value	HR	P value	HR	P value	HR	P value
	(95% CI)		(95% CI)		(95% CI)		(95% CI)	
Gender	0.05 (0 - 10.8)	0.72	0.05 (0 - 15.0)	0.726	/	0.839	/	0.613
Age	1.1 (1.0 - 1.3)	0.138	1.0 (0.912 - 1.2)	0.564	/	0.070	/	0.111
Smoking	0.73 (0.1 - 4.0)	0.713	0.2 (0 - 16.2)	0.252	/	0.979	/	0.301
Alcohol	3.2 (10.6 - 17.8)	0.176	7.4 (0.9 - 64.0)	0.069	/	0.194	/	0.147
Tumor location	3.7 (0.7 - 20.8)	0.135	6.1 (1.028 - 35.9)	**0.047**	/	0.108	/	0.951
Maximum tumor diameter	1.2 (0.6 - 2.4)	0.602	1.2 (0.708 - 2.2)	0.454	/	0.286	/	0.768
Tumor stage	2.0 (0.9 - 4.2)	0.069	3.1 (1.147 - 8.5)	**0.026**	/	0.373	/	0.622
Histological grade	1.0 (0.2 - 5.4)	1.00	1.1 (0.178 - 6.4)	0.944	/	0.326	/	0.866
Depth of submucosal invasion	1.6 (0.6 - 4.8)	0.37	2.3 (0.66 - 8.3)	0.188	/	0.926	/	0.625
Angiolymphatic invasion	4.1 (0.7 - 24.8)	0.126	6.5 (1.075 - 39.0)	**0.041**	/	0.614	/	0.552
Postoperative ctDNA status	27.5 (2.8 - 273.1)	**0.005**	27.6 (2.9 - 259.1)	**0.004**	184.6 (3.6–9576.9)	**0.01**	25.8 (2.7–242.6)	**0.004**

ctDNA, circulating tumor DNA; DFS, disease-free survival; OS, overall survival. The bold values indicate statistically significant differences.

## Discussion

In this study, we applied capture-based NGS to a panel consisting of 61 cancer-related genes of clinical significance to detect the matching FFPE tissues, pre- and postoperative plasma cfDNA, and WBC samples from 53 patients with ESCC. Our study showed that *TP53* and *PIK3CA* were frequently altered in patients with ESCC. Somatic mutations were detectable in pre-surgical plasma cfDNA of patients with stage 0-III ESCC, and at a lower MAF in several post-surgery cfDNA samples. This demonstrated that ctDNA detection after ESCC resection indicated MRD and identifies patients at high risk of recurrence and death.

We found that *TP53* and *PIK3CA* were the two most prominent mutated genes in the FFPE samples. The mutation rate of *TP53* in tumor samples was only 58.49%, which is lower than reported mutation frequency, mainly due to panel coverage (not all exons of *TP53* were included) and small sample size. The mutational spectrum of *TP53* is diverse in human cancers, apart from the “hotspot mutations” (23). The reported mutation rate of ESCC varies in different studies, ranging from 20% to 93%, which is mainly determined according to the sequencing panel coverage, methods used, and tumor stage (23–29). We identified more variants in pre-surgical cfDNA specimens compared with tumors, indicating spatial intratumoral heterogeneity in ESCC; Hao et al. also illustrated this (30).

Postoperative adjuvant therapy is recommended to eliminate micrometastases and MRD (31). Although in recent decades, an increasing number of immunohisto- or cytochemical and nucleic acid-based procedures have been developed, it has been reported that the occult tumor cell detection rates for lymph nodes of patients with esophageal cancer without overt lymph node metastases (pN0) was 26% to 56% (32, 33). Therefore, there is no reliable tool to assess MRD to tailor adjuvant therapy as early as possible. In addition, for ESCC, there is no effective tool to monitor early relapse without imaging. Previous studies have illustrated that ctDNA can be detected in most advanced-stage cancer patients with high sensitivity (34). Detection of ctDNA after resection can indicate MRD or even predict clinical recurrence and poor outcomes for different types of cancers (35, 36). Early detection of tumor relapse and a tool to evaluate treatment efficacy in ESCC would allow us to optimize individualized therapeutic strategies for patients. In the absence of effective prognostic biomarkers in ESCC (37), analysis of cfDNA may provide an easily accessible source of information to supervise tumor burden after surgery.

In our study, we detected a series of tumor-specific somatic mutations in the preoperative plasma cfDNA for the majority of patients with stage 0 to III ESCC using as little as 3 ml of blood. The overall sensitivity of *TP53* and *PIK3CA* detection in plasma cfDNA was 70%, which is lower than the detection sensitivity reported for lung cancer (19). The median MAF of pre-surgical plasma cfDNA variants was 0.17%, which was also lower than that reported in lung cancer, even though we utilized the same NGS assay (19). The sensitivity of cfDNA assays depends not only on preanalytical and analytical factors but also on the rate at which ctDNA is released from the tumor, the so-called “ctDNA shed” (38). Considering that we used the same plasma detection panel (Accu-Act) and platform (Firefly) as Xu et al. (19), tumor biology may determine sensitivity. Low tumor load may be one of the reasons why there are few successful MRD reports in ESCC compared to lung cancer or colorectal cancer. It has also been shown that gastroesophageal ctDNA has a relatively low number of mutant fragments (34).

In our study, six patients (three with stage IIB, two with stage IIIA, and one with IIIB) had detectable tumor-matching variants in their post-surgical cfDNA samples, with a relapse rate of 83.3%. In the six ctDNA+ patients, 50% had residual *TP53* mutations. However, our data showed that tissue P53 IHC status and *TP53* mutation status of tumor samples did not affect the prognosis (**Supplementary Figure 1**). However, 50% (3/6) of the ctDNA+ cases had residual *TP53* mutations. This might indicate that monitoring the changes in *TP53* mutations *via* plasma cfDNA is a potential approach to predict MRD or recurrence. Some cases in Ueda’s study demonstrated this possibility (9). In addition, this requires the detection of all *TP53* exons to ensure the detection of more cases.

Among the patients who were not treated with adjuvant therapy, four were cfDNA+, which demonstrated 100% sensitivity to predict recurrence, and 75% (3/4) of the patients relapsed within one year. This finding also demonstrates that postoperative plasma cfDNA positivity is an independent disadvantage for patients with ESCC. Hsieh et al. revealed that higher cfDNA levels are associated with tumor relapse and shorter DFS after esophagectomy in patients with ESCC (39). Recently, a study described that ctDNA detection in patients who received chemoradiotherapy without surgery is associated with tumor progression and disease-specific survival, with predicted progression in 71% of patients. Another study also indicates that ctDNA detection post-chemoradiotherapy can predict tumor progression by an average of 2.8 months earlier than PET-CT imaging (40). Furthermore, adjuvant chemotherapy (ACT) can eliminate the residual disease in up to 30% of postoperative ctDNA+ patients, and therefore, can be a treatment option for ctDNA+ patients with CRC (41). Both studies and our results illustrate that ctDNA analysis could be used to identify patients at high risk for tumor progression.

Moreover, Zhang et al. explored the cfDNA mutation signature of patients with ESCC who were responsive and non-responsive to neoadjuvant chemotherapy (NAC). It was illustrated that the driver gene molecular mutation burden (MMB) of the responsive group was significantly higher than that of the non-responsive group and that the plasma cfDNA MMB and CNVs may be used to predict the response of ESCC patients to NAC (42). Likewise, a study with a cohort of patients with early breast cancer who received NAC found that ctDNA levels after two cycles of NAC predict the local tumor response to NAC treatment, and positive baseline ctDNA is significantly associated with worse DFS and OS (43). In colorectal cancer, ctDNA analysis of post-chemotherapy plasma revealed that ctDNA+ patients were more than 40 times more likely to experience disease recurrence than ctDNA- patients (17). Raja et al. demonstrated that early reduction in ctDNA MAF may be a useful predictor of long-term benefit from immunotherapy in patients with lung and bladder cancer, and the decrease in mean MAF may precede radiographic change in tumor volume by one year (44). Pretreatment ctDNA levels also appear to be prognostic and help predict the dynamics of durvalumab treatment in 16 advanced tumor types as reported in a previous study (45). In patients with urothelial bladder carcinoma, the ctDNA-NGS approach could predict metastatic relapse and monitor therapeutic efficacy (46). Furthermore, plasma ctDNA is a potential biomarker for multiple cancers with high sensitivity and specificity (47–50).

There are two strategies to monitor tumor MRD: Tumor-informed (fixed or personalized) assay and tumor-agnostic approach (also called tumor-naïve or plasma-only approach). The former approach, based on tumor sequencing, can accurately filter non-tumor-derived mutations, such as germline mutations and clonal hematopoiesis (CH). The fixed tumor-informed assay utilized in the present study may lead to a certain percentage of false negatives, even though it is moderately cost-effective and has a decent turn-around time (TAT). Signatera demonstrated that a personalized tumor-informed assay can improve sensitivity and specificity in lung cancer, CRC, and breast cancer (51, 52). Compared with the fixed strategy, it can help the patient save 42% (lead time 264 *vs* 187 days) more time to adopt a suitable treatment (51). However, the clinical application of this approach could be limited due to the unavailability of sufficient tumor samples, long development period of a personalized panel for each patient, and high cost. The tumor-agnostic approach represented by Guardant Reveal only requires plasma cfDNA sequencing using a fixed panel; it reduces cost and TAT and is more suitable for clinical application. The disadvantage of this method is the lack of sensitivity, which can be promoted by analyzing multiple biomarkers, like ctDNA mutation and ctDNA methylation. The potential shortcoming of the tumor-agnostic approach is that it may result in false positives when an inappropriate variant filter strategy is used. In the present study, we used a tumor-informed assay with a fixed panel, which showed 60% sensitivity and 95.45% specificity in the cohort that did not receive adjuvant therapy, as well as a tumor-agnostic approach in the same cohort, which showed 80% sensitivity and 63.64% specificity (**Supplementary Table S7**). Interestingly, we found some TP53 variants derived from CH. Mutations originating from CH could lead to faulty reporting and incorrect treatment strategies when tumor-only sequencing is used (53). An increasing number of studies confirm that *TP53* mutation has a prognostic value in esophageal cancer (23, 54, 55), attention should be paid to the CH phenomenon. cfDNA-leukocyte paired sequencing is essential to accurately identify the source of the genetic variation, and more attempts and efforts are still needed to evaluate MRD in esophageal cancer.

Even though the sample size in our study was not large enough, this is the first study to date to analyze pre-surgical and post-surgical cfDNA in more than 50 patients with stage 0-III ESCC. However, other studies have mainly concentrated on patients with stage II-III ESCC (9, 56). Accurate detection of MRD can guide individual and personalized management of patients with early-stage ESCC. As MRD detection after surgical resection remains challenging, there is still much work to do. Limitations of this study are that blood collection time points may seem inadequate, as longitudinal blood collection at multiple time points (including time points before and after adjuvant therapy) was also required to demonstrate how early ctDNA indicates relapse and the extent to which adjuvant therapy can eliminate the residual disease in patients with ESCC. We could not explore the potential value of ctDNA at the time of adjuvant treatment because of the lack of sample availability. We have ongoing studies (ChiCTR2000034355) to explore these. Second, this study was initiated in 2015, and the postoperative blood collection time was one week after surgery. With the improvement of cfDNA research, it has been shown in multiple studies that there is an initial spike in cfDNA following surgery due to trauma-induced cfDNA, and it takes 3 to 4 weeks for cfDNA levels to return to stasis (57). Trauma causes increased cell death and has been associated with increased levels of wild-type cfDNA in the blood. This would cause a low ctDNA content in the circulation, increasing the limit of detection for the ctDNA detection (17, 57, 58). In this study, we found 15 patients whose cfDNA concentration post-surgery was higher than that before surgery (**Supplementary Table S1**). To reduce the impact of trauma-induced cfDNA on ctDNA detection, a distant timepoint, such as 3 to 4 weeks, may be more appropriate for reliable MRD detection in solid tumors. We have noticed this before and used one month (± 1 week) as the first time point post-surgery in our ongoing study. Third, the sensitivity of our NGS assay was lower than that reported in a previous study, possibly due to limitations in the NGS panel we selected. High alteration rate genes identified by WGS/WES, such as *KMT2D, FAT1, FAT3, NRF2, and EP300* (24, 59) were not included in our panel. At the same time, we should consider the cost of sequencing, as the detection rate was 83% even though a 483 cancer-related gene-containing panel was used (14). The gene panel needs to be optimized and specified for ESCC in future research. Fourth, despite the relatively larger number of patients in this study, our conclusions are still limited by the small number of postoperative ctDNA+ patients; further large-scale trials are still needed.

In conclusion, the results of our study indicate that ctDNA analysis is a valuable method for risk stratification of patients with resectable ESCC. Specifically, the detection of ctDNA variants in postoperative plasma is strongly prognostic for DFS and OS.

## Data Availability Statement

The raw sequencing data presented in this study is deposited in the China National Genebank (CNGB, https://db.cngb.org/cnsa/), the accession number is CNP0001778. According to national legislation, specifically the Administrative Regulations of the People's Republic of China on Human Genetic Resources (http://www.gov.cn/zhengce/content/2019-06/10/content_5398829.htm, http://english.www.gov.cn/policies/latest_releases/2019/06/10/content_281476708945462.htm), no additional raw data is available at this time. Data of this project can be accessed after an approval application to the CNGB. Please email: CNGBdb@cngb.org for detailed application guidance. The accession code CNP0001778 should be included in the application.

## Ethics Statement

The studies involving human participants were reviewed and approved by the institutional review board of Changhai Hospital. The patients/participants provided their written informed consent to participate in this study.

## Author Contributions

TL contributed to sample preparation, collection of all clinical information of patients, follow-up of patients, and assisting in the writing of manuscripts. QY was in-charge of writing the manuscript. HJ contributed to experimental design and organization, analysis with constructive discussions, and paper modification. All authors contributed to the article and approved the submitted version.

## Funding

This study was supported by the Shanghai Science and Technology Committee (no. 15411951700).

## Conflict of Interest

QY was employed by Shanghai AccuraGen Biotechnology Co., Ltd.

The remaining authors declare that the research was conducted in the absence of any commercial or financial relationships that could be construed as a potential conflict of interest.

## References

[1] Siegel RL Miller KD Jemal A. Cancer Statistics, 2017. CA Cancer J Clin (2017) 67(1):7–30. 10.3322/caac.21387 28055103

[2] Hiyama T Yoshihara M Tanaka S Chayama K. Genetic Polymorphisms and Esophageal Cancer Risk. Int J Cancer (2007) 121(8):1643–58. 10.1002/ijc.23044 17674367

[3] Yang H Liu H Chen Y Zhu C Fang W Yu Z. Neoadjuvant Chemoradiotherapy Followed by Surgery Versus Surgery Alone for Locally Advanced Squamous Cell Carcinoma of the Esophagus (Neocrtec5010): A Phase III Multicenter, Randomized, Open-Label Clinical Trial. J Clin Oncol (2018) 36(27):2796–803. 10.1200/JCO.2018.79.1483 PMC6145832 30089078

[4] Su X-D Zhang D-K Zhang X Lin P Long H Rong T-H. Prognostic Factors in Patients With Recurrence After Complete Resection of Esophageal Squamous Cell Carcinoma. J Thorac Dis (2014) 6(7):949–57. 10.3978/j.issn.2072-1439.2014.07.14 PMC4120168 25093092

[5] Xu Y Chen Q Yu X Zhou X Zheng X Mao W. Factors Influencing the Risk of Recurrence in Patients With Esophageal Carcinoma Treated With Surgery: A Single Institution Analysis Consisting of 1002 Cases. Oncol Lett (2013) 5(1):185–90. 10.3892/ol.2012.1007 PMC3525364 23255917

[6] Scheuemann P Hosch SB Izbicki JR. Prognostic Value of Minimal Residual Disease in Esophageal Cancer. In: Pantel K, editor. Micrometastasis. Cancer Metastasis - Biology and Treatment, vol. 5. Dordrecht: Springer (2003). p. 127–38. 10.1007/978-1-4020-4460-1_7

[7] Lagergren J. Adenocarcinoma of Oesophagus: What Exactly is the Size of the Problem and Who is At Risk? Gut (2005) 54 Suppl 1:i1–5. 10.1136/gut.2004.041517 PMC1867797 15711002

[8] Komatsu S Ichikawa D Hirajima S Takeshita H Shiozaki A Fujiwara H. Clinical Impact of Predicting CCND1 Amplification Using Plasma DNA in Superficial Esophageal Squamous Cell Carcinoma. Dig Dis Sci (2014) 59(6):1152–9. 10.1007/s10620-013-3005-2 24458211

[9] Ueda M Iguchi T Masuda T Nakahara Y Hirata H Uchi R. Somatic Mutations in Plasma Cell-Free DNA are Diagnostic Markers for Esophageal Squamous Cell Carcinoma Recurrence. Oncotarget (2016) 7(38):62280–91. 10.18632/oncotarget.11409 PMC5308726 27556701

[10] Koppert LB Wijnhoven BP van Dekken H Tilanus HW Dinjens WN. The Molecular Biology of Esophageal Adenocarcinoma. J Surg Oncol (2005) 92(3):169–90. 10.1002/jso.20359 16299787

[11] Vallbohmer D Lenz HJ. Predictive and Prognostic Molecular Markers in Outcome of Esophageal Cancer. Dis Esophagus (2006) 19(6):425–32. 10.1111/j.1442-2050.2006.00622.x 17069584

[12] Diaz LA Jr Bardelli A. Liquid Biopsies: Genotyping Circulating Tumor DNA. J Clin oncology: Off J Am Soc Clin Oncol (2014) 32(6):579–86. 10.1200/JCO.2012.45.2011 PMC4820760 24449238

[13] Diehl F Schmidt K Choti MA Romans K Goodman S Li M. Circulating Mutant DNA to Assess Tumor Dynamics. Nat Med (2008) 14(9):985–90. 10.1038/nm.1789 PMC2820391 18670422

[14] Meng P Wei J Geng Y Chen S Terpstra MM Huang Q. Targeted Sequencing of Circulating Cell-Free DNA in Stage II-III Resectable Oesophageal Squamous Cell Carcinoma Patients. BMC Cancer (2019) 19(1):818. 10.1186/s12885-019-6025-2 31429737 PMC6701116

[15] Chaudhuri AA Chabon JJ Lovejoy AF Newman AM Stehr H Azad TD. Early Detection of Molecular Residual Disease in Localized Lung Cancer by Circulating Tumor DNA Profiling. Cancer Discovery (2017) 7(12):1394–403. 10.1158/2159-8290.CD-17-0716 PMC5895851 28899864

[16] Garcia-Murillas I Schiavon G Weigelt B Ng C Hrebien S Cutts RJ. Mutation Tracking in Circulating Tumor DNA Predicts Relapse in Early Breast Cancer. Sci Transl Med (2015) 7(302):302ra133. 10.1126/scitranslmed.aab0021 26311728

[17] Reinert T Henriksen TV Christensen E Sharma S Salari R Sethi H. Analysis of Plasma Cell-Free DNA by Ultradeep Sequencing in Patients With Stages I to III Colorectal Cancer. JAMA Oncol (2019) 5(8):1124–31. 10.1001/jamaoncol.2019.0528 PMC6512280 31070691

[18] Gao XH Li J Gong HF Yu GY Liu P Hao LQ. Comparison of Fresh Frozen Tissue With Formalin-Fixed Paraffin-Embedded Tissue for Mutation Analysis Using a Multi-Gene Panel in Patients With Colorectal Cancer. Front Oncol (2020) 10:310–0. 10.3389/fonc.2020.00310 PMC7083147 32232001

[19] Xu T Kang X You X Dai L Tian D Yan W. Cross-Platform Comparison of Four Leading Technologies for Detecting Egfr Mutations in Circulating Tumor DNA From Non-Small Cell Lung Carcinoma Patient Plasma. Theranostics (2017) 7(6):1437–46. 10.7150/thno.16558 PMC5436504 28529628

[20] Li H Durbin R. Fast and Accurate Long-Read Alignment With Burrows–Wheeler Transform. Bioinformatics (2010) 26(5):589–95. 10.1093/bioinformatics/btp698 PMC2828108 20080505

[21] Lai Z Markovets A Ahdesmaki M Chapman B Hofmann O McEwen R. VarDict: A Novel and Versatile Variant Caller for Next-Generation Sequencing in Cancer Research. Nucleic Acids Res (2016) 44(11):e108. 10.1093/nar/gkw227 27060149 PMC4914105

[22] Paila U Chapman BA Kirchner R Quinlan AR. GEMINI: Integrative Exploration of Genetic Variation and Genome Annotations. PloS Comput Biol (2013) 9(7):e1003153. 10.1371/journal.pcbi.1003153 23874191 PMC3715403

[23] Huang M Jin J Zhang F Wu Y Xu C Ying L. Non-Disruptive Mutation in TP53 DNA-Binding Domain is a Beneficial Factor of Esophageal Squamous Cell Carcinoma. Ann Transl Med (2020) 8(6):316. 10.21037/atm.2020.02.142 32355760 PMC7186752

[24] Gao YB Chen ZL Li JG Hu XD Shi XJ Sun ZM. Genetic Landscape of Esophageal Squamous Cell Carcinoma. Nat Genet (2014) 46(10):1097–102. 10.1038/ng.3076 25151357

[25] Soussi T Legros Y Lubin R Ory K Schlichtholz B. Multifactorial Analysis of p53 Alteration in Human Cancer: A Review. Int J Cancer (1994) 57(1):1–9. 10.1002/ijc.2910570102 8150526

[26] Li XC Wang MY Yang M Dai HJ Zhang BF Wang W. A Mutational Signature Associated With Alcohol Consumption and Prognostically Significantly Mutated Driver Genes in Esophageal Squamous Cell Carcinoma. Ann Oncol (2018) 29(4):938–44. 10.1093/annonc/mdy011 PMC5913594 29351612

[27] Hu N Huang J Emmert-Buck MR Tang ZZ Roth MJ Wang C. Frequent Inactivation of the TP53 Gene in Esophageal Squamous Cell Carcinoma From a High-Risk Population in China. Clin Cancer Res (2001) 7(4):883–91. 11309337

[28] Hainaut P Hollstein M. p53 and Human Cancer: The First Ten Thousand Mutations. Adv Cancer Res (2000) 77:81–137. 10.1016/S0065-230X(08)60785-X 10549356

[29] Zheng H Wang Y Tang C Jones L Ye H Zhang G. Tp53, PIK3CA, FBXW7 and KRAS Mutations in Esophageal Cancer Identified by Targeted Sequencing. Cancer Genomics Proteomics (2016) 13(3):231–8. PMC7495501 27107065

[30] Hao JJ Lin DC Dinh HQ Mayakonda A Jiang YY Chang C. Spatial Intratumoral Heterogeneity and Temporal Clonal Evolution in Esophageal Squamous Cell Carcinoma. Nat Genet (2016) 48(12):1500–7. 10.1038/ng.3683 PMC5127772 27749841

[31] Li Q Luo D Zhu J Yang L Liu Q Ma Y. AcrnaCT Trial Protocol: Efficacy of Adjuvant Chemotherapy in Patients With Clinical T3b/T4, N+ Rectal Cancer Undergoing Neoadjuvant Chemoradiotherapy: A Pathology-Oriented, Prospective, Multicenter, Randomized, Open-Label, Parallel Group Clinical Trial. BMC Cancer (2019) 19(1):1117. 10.1186/s12885-019-6289-6 31729964 PMC6858777

[32] Matsumoto M Natsugoe S Nakashima S Sakamoto F Okumura H Sakita H. Clinical Significance of Lymph Node Micrometastasis of pN0 Esophageal Squamous Cell Carcinoma. Cancer Lett (2000) 153(1-2):189–97. 10.1016/S0304-3835(00)00374-8 10779649

[33] Hosch S Kraus J Scheunemann P Izbicki JR Schneider C Schumacher U. Malignant Potential and Cytogenetic Characteristics of Occult Disseminated Tumor Cells in Esophageal Cancer. Cancer Res (2000) 60(24):6836–40. 11156375

[34] Bettegowda C Sausen M Leary RJ Kinde I Wang Y Agrawal N. Detection of Circulating Tumor DNA in Early- and Late-Stage Human Malignancies. Sci Transl Med (2014) 6(224):224ra24. 10.1158/1538-7445.AM2014-5606 PMC4017867 24553385

[35] Sausen M Phallen J Adleff V Jones S Leary RJ Barrett MT. Clinical Implications of Genomic Alterations in the Tumour and Circulation of Pancreatic Cancer Patients. Nat Commun (2015) 6:7686. 10.1158/1538-7445.AM2015-619 26154128 PMC4634573

[36] Majure M Logan AC. What the Blood Knows: Interrogating Circulating Tumor DNA to Predict Progression of Minimal Residual Disease in Early Breast Cancer. Ann Transl Med (2016) 4(24):543. 10.21037/atm.2016.11.77 28149904 PMC5233500

[37] Qing T Zhu S Suo C Zhang L Zheng Y Shi L. Somatic Mutations in ZFHX4 Gene are Associated With Poor Overall Survival of Chinese Esophageal Squamous Cell Carcinoma Patients. Sci Rep (2017) 7(1):4951. 10.1038/s41598-017-04221-7 28694483 PMC5504002

[38] Cho MS Park CH Lee S Park HS. Clinicopathological Parameters for Circulating Tumor DNA Shedding in Surgically Resected non-Small Cell Lung Cancer With EGFR or KRAS Mutation. PloS One (2020) 15(3):e0230622. 10.1371/journal.pone.0230622 32196518 PMC7083310

[39] Hsieh CC Hsu HS Chang SC Chen YJ. Circulating Cell-Free Dna Levels Could Predict Oncological Outcomes of Patients Undergoing Esophagectomy for Esophageal Squamous Cell Carcinoma. Int J Mol Sci (2016) 17(12):2131. 10.3390/ijms17122131 PMC5187931 27999323

[40] Azad TD Chaudhuri AA Fang P Qiao Y Esfahani MS Chabon JJ. Circulating Tumor Dna Analysis for Detection of Minimal Residual Disease After Chemoradiotherapy for Localized Esophageal Cancer. Gastroenterology (2020) 158(3):494–505.e6. 10.1053/j.gastro.2019.10.039 31711920 PMC7010551

[41] Reinert T Henriksen TV Rasmussen MH Sethi H Salari R Shchegrova S. Serial Circulating Tumor DNA Analysis for Detection of Residual Disease, Assessment of Adjuvant Therapy Efficacy and for Early Recurrence Detection in Colorectal Cancer. Ann Oncol (2018) 29:viii151. 10.1093/annonc/mdy281.004

[42] Zhang R Hu Y Zhou T Han W Liu Y Qian J. The Mutation Profiles of Cell-Free DNA in Patients With Oesophageal Squamous Cell Carcinoma Who Were Responsive and non-Responsive to Neoadjuvant Chemotherapy. J Thorac Dis (2020) 12(8):4274–83. 10.21037/jtd-20-230 PMC7475527 32944339

[43] Li S Lai H Liu J Liu Y Jin L Li Y. Circulating Tumor Dna Predicts the Response and Prognosis in Patients With Early Breast Cancer Receiving Neoadjuvant Chemotherapy. JCO Precis Oncol (2020) 4:244–57. 10.1200/PO.19.00292 PMC7450928 32923909

[44] Raja R Kuziora M Brohawn PZ Higgs BW Gupta A Dennis PA. Early Reduction in Ctdna Predicts Survival in Patients With Lung and Bladder Cancer Treated With Durvalumab. Clin Cancer Res (2018) 24(24):6212–22. 10.1158/1078-0432.CCR-18-0386 30093454

[45] Zhang Q Luo J Wu S Si H Gao C Xu W. Prognostic and Predictive Impact of Circulating Tumor DNA in Patients With Advanced Cancers Treated With Immune Checkpoint Blockade. Cancer Discovery (2020) 10(12):1842. 10.1158/2159-8290.CD-20-0047 32816849 PMC8358981

[46] Christensen E Birkenkamp-Demtröder K Sethi H Shchegrova S Salari R Nordentoft I. Early Detection of Metastatic Relapse and Monitoring of Therapeutic Efficacy by Ultra-Deep Sequencing of Plasma Cell-Free DNA in Patients With Urothelial Bladder Carcinoma. J Clin Oncol (2019) 37(18):1547–57. 10.1158/1538-7445.AM2019-913 31059311

[47] Cristiano S Leal A Phallen J Fiksel J Adleff V Bruhm DC. Genome-Wide Cell-Free DNA Fragmentation in Patients With Cancer. Nature (2019) 570(7761):385–9. 10.1038/s41586-019-1272-6 PMC6774252 31142840

[48] Wang Y Li L Douville C Cohen JD Yen T-T Kinde I. Evaluation of Liquid From the Papanicolaou Test and Other Liquid Biopsies for the Detection of Endometrial and Ovarian Cancers. Sci Trans Med (2018) 10(433):eaap8793. 10.1126/scitranslmed.aap8793 PMC6320220 29563323

[49] Qu C Wang Y Wang P Chen K Wang M Zeng H. Detection of Early-Stage Hepatocellular Carcinoma in Asymptomatic HBsAg-seropositive Individuals by Liquid Biopsy. Proc Natl Acad Sci USA (2019) 116(13):6308–12. 10.1073/pnas.1819799116 PMC6442629 30858324

[50] Cohen JD Li L Wang Y Thoburn C Afsari B Danilova L. Detection and Localization of Surgically Resectable Cancers With a Multi-Analyte Blood Test. Science (2018) 359(6378):926–30. 10.1126/science.aar3247 PMC6080308 29348365

[51] Coombes RC Page K Salari R Hastings RK Armstrong A Ahmed S. Personalized Detection of Circulating Tumor DNA Antedates Breast Cancer Metastatic Recurrence. Clin Cancer Res (2019) 25(14):4255–63. 10.1158/1078-0432.CCR-18-3663 30992300

[52] Oliveira KCS Ramos IB Silva JMC Barra WF Riggins GJ Palande V. Current Perspectives on Circulating Tumor DNA, Precision Medicine, and Personalized Clinical Management of Cancer. Mol Cancer Res (2020) 18(4):517–28. 10.1158/1541-7786.MCR-19-0768 31996469

[53] Ptashkin RN Mandelker DL Coombs CC Bolton K Yelskaya Z Hyman DM. Prevalence of Clonal Hematopoiesis Mutations in Tumor-Only Clinical Genomic Profiling of Solid Tumors. JAMA Oncol (2018) 4(11):1589–93. 10.1001/jamaoncol.2018.2297 PMC6224316 29872864

[54] Fisher OM Lord SJ Falkenback D Clemons NJ Eslick GD Lord RV. The Prognostic Value of TP53 Mutations in Oesophageal Adenocarcinoma: A Systematic Review and Meta-Analysis. Gut (2017) 66(3):399–410. 10.1136/gutjnl-2015-310888 26733670 PMC5534764

[55] Zhang D Zhang W Liu W Mao Y Fu Z Liu J. Human Papillomavirus Infection Increases the Chemoradiation Response of Esophageal Squamous Cell Carcinoma Based on P53 Mutation. Radiotherapy Oncol (2017) 124(1):155–60. 10.1016/j.radonc.2017.06.008 28647401

[56] Andolfo I Petrosino G Vecchione L De Antonellis P Capasso M Montanaro D. Detection of erbB2 Copy Number Variations in Plasma of Patients With Esophageal Carcinoma. BMC Cancer (2011) 11:126. 10.1186/1471-2407-11-126 21481261 PMC3094322

[57] Henriksen TV Reinert T Christensen E Sethi H Birkenkamp-Demtröder K Gögenur M. The Effect of Surgical Trauma on Circulating Free DNA Levels in Cancer Patients-Implications for Studies of Circulating Tumor DNA. Mol Oncol (2020) 14(8):1670–9. 10.1002/1878-0261.12729 PMC7400779 32471011

[58] Ignatiadis M Lee M Jeffrey SS. Circulating Tumor Cells and Circulating Tumor DNA: Challenges and Opportunities on the Path to Clinical Utility. Clin Cancer Res (2015) 21(21):4786–800. 10.1158/1078-0432.CCR-14-1190 26527805

[59] Sawada G Niida A Uchi R Hirata H Shimamura T Suzuki Y. Genomic Landscape of Esophageal Squamous Cell Carcinoma In a Japanese Population. Gastroenterology (2016) 150(5):1171–82. 10.1053/j.gastro.2016.01.035 26873401

