# Supplementary material for: Plasma Circulating Tumor DNA Sequencing Predicts Minimal Residual Disease in Resectable Esophageal Squamous Cell Carcinoma
Source: Front Oncol. 2021 May 20;11:616209. doi: 10.3389/fonc.2021.616209 (PMC8173109; doi:10.3389/fonc.2021.616209)
Supplement: Supplementary file 2 [file DataSheet_2.docx]

Supplementary Material

# Supplementary Data

**TP53 and survival**

As *TP53* is the gene with the highest mutation rate in ESCC, we explored its value in predicting patient prognosis. Primarily, we stratified patients according to the expression status of P53 (positive, P53+; negative, P53-) identified by IHC (Supplementary Table S8). The P53+ patients had a median DFS of 32.2 months versus a median DFS of 32.6 months in TP53- patients, which showed no statistical difference (**Figure S2A**). P53+ patients had a median OS of 39.5 months versus not reach for P53- patients, but the difference was not statistically significant (HR: 0.803; 95% CI, 0.34 – 1.896; P = 0.617; **Figure S2B**). Then, we used the tumor FFPE mutation status to group the subjects, the *TP53* mutation (TP53-mut) patients had a median DFS of 29.8 months versus median DFS of 32.6 months in wildtype (TP53-wt) patients, no statistical difference (P = 0.834; **Figure S2C**). TP53-wt patients had a median OS of 39.5 months versus not reach for TP53-mut patients, but the difference was not statistically significant (P = 0.746; **Figure S2D**). It showed that *TP53* IHC status and tumor mutation status were not predictive factors for the survival of ESCC patients.

# Supplementary Figures and Tables


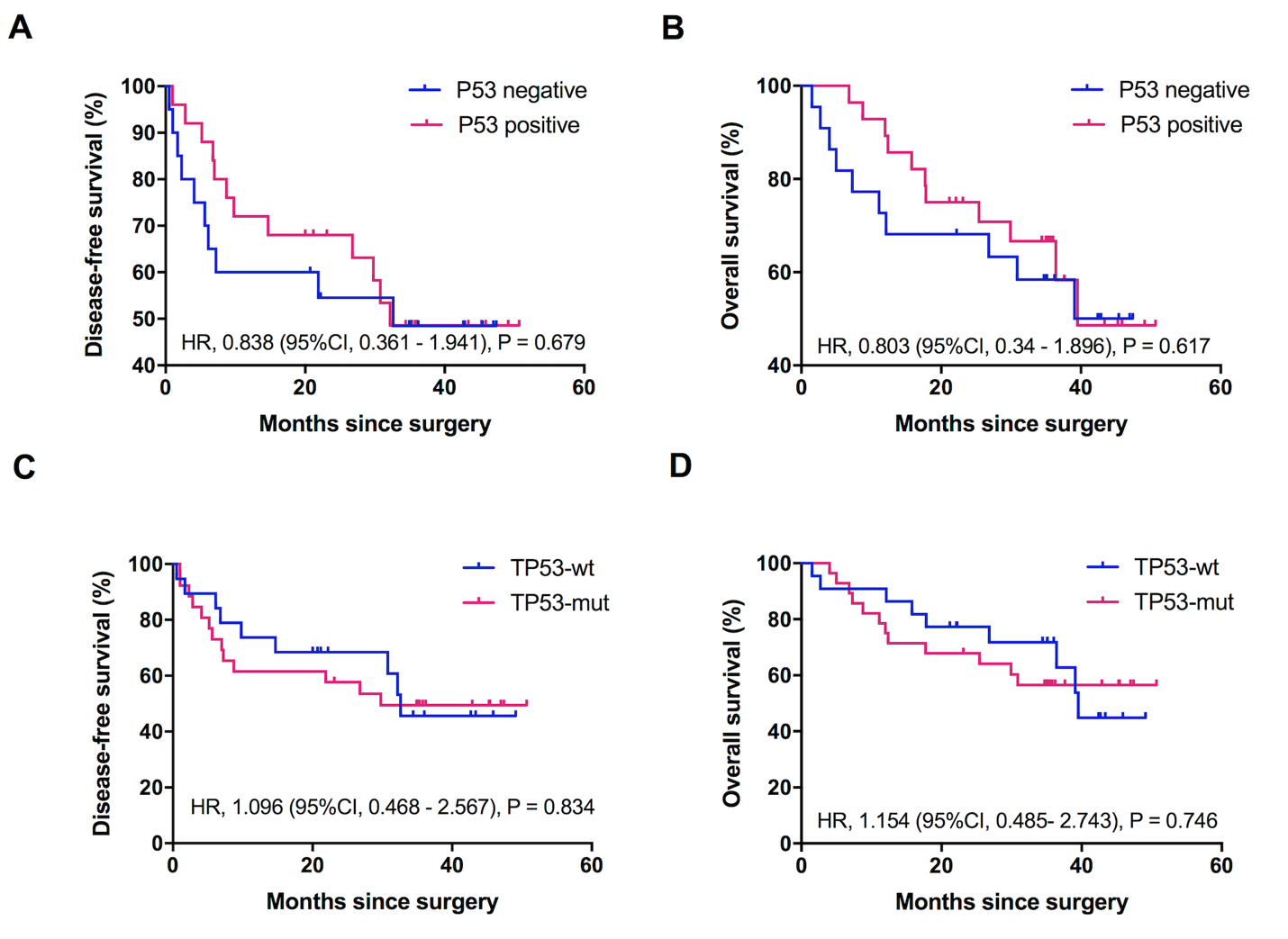


**Supplementary Figure 2.** **TP53 status and prognosis of patients with ESCC.**

(A) Kaplan–Meier survival curves for DFS analysis between p53 expression status detected by IHC. (B) Kaplan–Meier survival curves for OS analysis between p53 positive and negative groups detected by IHC. (C) Kaplan-Meier estimates of DFS according to tumor *TP53* mutation status. (D) Kaplan–Meier survival curves for OS analysis by tumor *TP53* mutation status.
